# MicroRNA cargo of extracellular vesicles as mediators of immune dysregulation in pediatric immune thrombocytopenia: from pathogenetic drivers to biomarkers

**DOI:** 10.3389/fgene.2026.1951187

**Published:** 2026-10-12

**Authors:** Lucia Argenziano, Maria Maddalena Marrapodi, Giuseppe Di Feo, Oriana Di Domenico, Francesca Rossi, Alessandra Di Paola

**Affiliations:** 1 Department of Woman, Child and General and Specialist Surgery, University of Campania “Luigi Vanvitelli”, Naples, Italy; 2 Department of Life Sciences, Health and Health Professions, Link Campus University, Rome, Italy

**Keywords:** biomarkers, extracellular vesicles, immune thrombocytopenia, microRNA, mir-146a

## Abstract

Pediatric immune thrombocytopenia (ITP) is an acquired autoimmune disorder, caused by the production of autoantibodies against platelets which leads to thrombocytopenia. It is characterized by profound alterations in both inflammatory and immune profiles. In this context, the role of microRNAs (miRNAs) as post-transcriptional regulators of immunity and the function of extracellular vesicles (EVs) as key mediators of intercellular communication are two fundamental aspects for understanding ITP pathophysiology. In ITP, altered miRNA expression disrupts immune tolerance, lymphocyte subtype differentiation and platelet production. miRNAs are incorporated into EVs and transferred to recipient cells, which confers greater stability to the miRNAs. To date, the interaction between miRNAs and EVs in pediatric ITP has been relatively understudied. In this review, we examine the landscape of dysregulated miRNAs in ITP, focusing in particular on their role in immune dysregulation and on the contribution of EVs as carriers of pathogenic miRNAs. The relationship between EVs and miRNAs could facilitate the development of non-invasive diagnostic tools and EV-based therapeutic strategies designed to reestablish immune homeostasis in pediatric ITP.

## 1 Introduction

Immune thrombocytopenia (ITP) is an acquired autoimmune disorder affecting children and adolescents aged between 1 and 18 years, which leads to the production of autoantibodies against platelets. This condition causes thrombocytopenia, with a platelet count below 100 × 10^9^/L and an increased risk of minor bleeding (Di Paola et al., 2022; Gotesman et al., 2024; Singh et al., 2021). Pediatric ITP is usually an acute, self-limiting condition, characterised by a higher rate of spontaneous remission, but around 20%–30% of patients progress to a chronic form requiring long-term management (Despotovic and Grimes, 2018; Lee, 2023; Thakur et al., 2024). In general, ITP is classified as newly diagnosed within 3 months of onset, persistent if it lasts between 3 and 12 months, and chronic if it lasts for more than 12 months (Provan et al., 2019). First-line therapy consists of a watch-and-wait approach in newly diagnosed ITP pediatric patients with mild or no bleeding (Despotovic and Grimes, 2018). When necessary, intravenous immunoglobulin (IVIG) and glucocorticosteroids, such as prednisone, methylprednisolone, or dexamethasone are administered. In cases of chronic ITP refractory to first-line therapies, combining first-line treatments or thrombopoietin receptor agonists (TPO-RAs), including romiplostim and eltrombopag, represent second-line treatment options (Russo et al., 2024). The therapeutic effect of TPO-RAs is based on the activation of the thrombopoietin (TPO) receptor, which promotes the proliferation and maturation of megakaryocytes and platelet production (Wang Y. et al., 2026). For patients who respond poorly to TPO-RAs, rituximab, an anti-CD20 monoclonal antibody, is commonly recommended as a therapeutic option, as it exerts its immunosuppressive activity through B-cell depletion (Russo et al., 2024; Bussel and Garcia, 2022). ITP patients exhibit profound alterations in both their inflammatory and immune profiles, with a prevalence of the pro-inflammatory M1 macrophage phenotype and a high ratio of pro-inflammatory T helper 1 cells/anti-inflammatory T helper 2 cells (Th1/Th2), resulting in impaired inflammatory profiles and immune responses. In addition, mesenchymal stromal cells (MSCs), multipotent non‐haematopoietic cells with anti‐inflammatory and immunomodulatory properties, are strongly defective in ITP (Di Paola et al., 2020; He et al., 2021) (Figure 1). MicroRNAs (miRNAs) are short non-coding RNA molecules (19–25 nt) that regulate gene expression by silencing specific target transcripts and widely recognized as key regulators of immune homeostasis (Zhao et al., 2022). Extracellular vesicles (EVs) are a population of heterogeneous, membrane-bound carriers containing complex cargo, such as proteins, lipids, and nucleic acids, including miRNAs, which can be released by a variety of cells under physiological or pathological conditions (Payandeh et al., 2024; Villar-Vesga et al., 2019). These mechanisms highlight the need for dedicated studies on EV-associated miRNA (EV-miRNAs). In this context, EVs derived from megakaryocytes, platelets, and stromal cells may contribute to immune regulation and platelet homeostasis, transferring functional miRNAs (D'Amico et al., 2025). Recent advances have highlighted two complementary aspects of ITP pathophysiology: the role of the miRNAs as post-transcriptional regulators of immunity, and the function of EVs as key mediators of intercellular communication. A recent review has exhaustively summarised the importance of miRNAs in ITP, placing particular focus on immune dysregulation, megakaryocytic insufficiency, the potential of biomarkers and functional evidence (Liu et al., 2026). However, how miRNAs and EVs interact remains an underexplored area in pediatric ITP. In this review, we explore the landscape of deregulated miRNAs in ITP, with particular attention to their role in immune dysregulation, and the contribution of EVs as carriers of pathogenic miRNAs. A deeper understanding of the EV-miRNA signature may represent a potential diagnostic and therapeutic tool in pediatric ITP.

**FIGURE 1:**
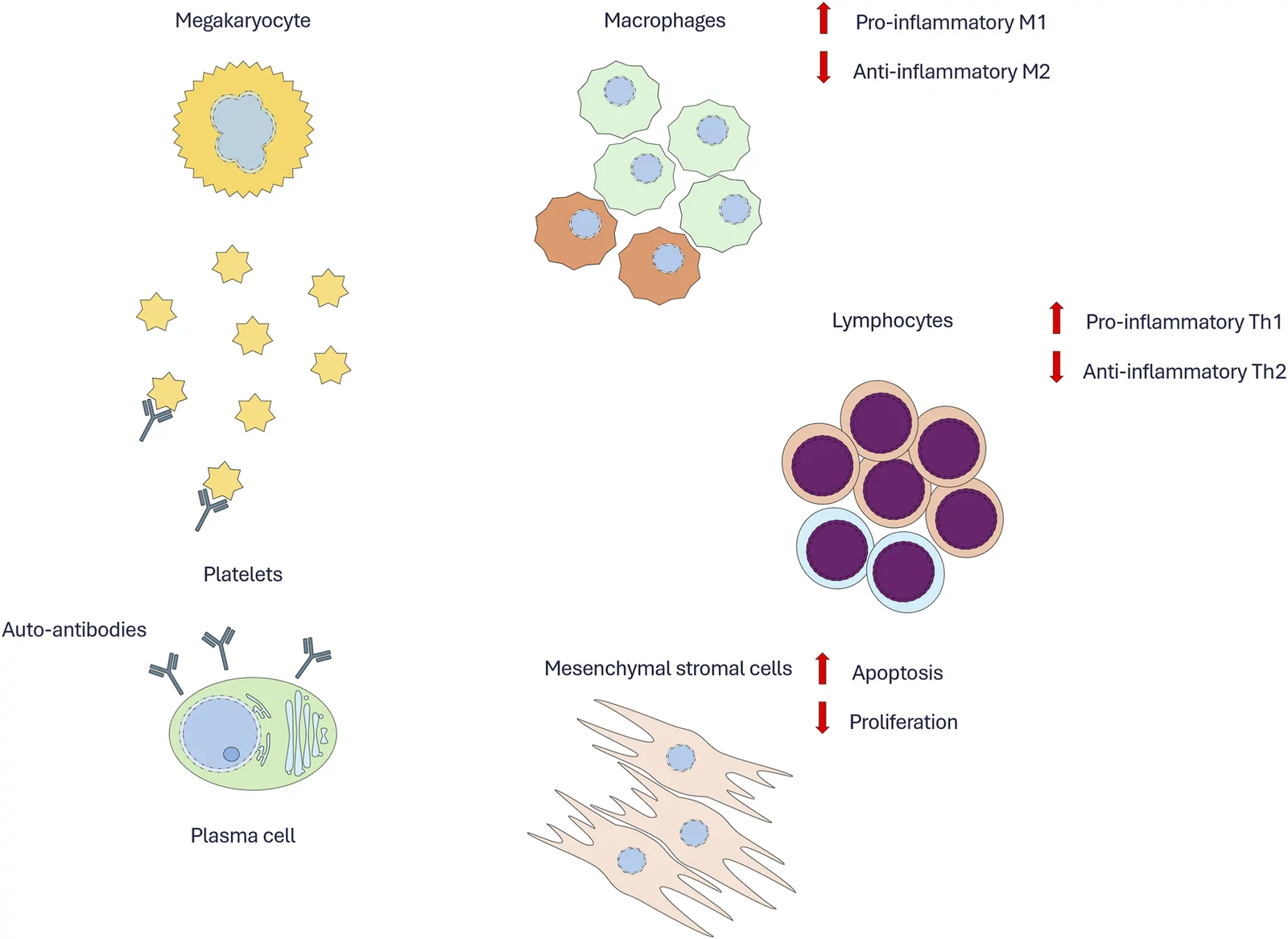
Pathophysiology of ITP disease. ITP patients exhibit profound alterations in inflammatory and immune profiles, caused by the production of autoantibodies against platelets which leads to thrombocytopenia. It is characterized by a prevalence of the pro-inflammatory M1 macrophage phenotype and a high ratio of Th1/Th2 lymphocytes, resulting in impaired inflammatory profiles and immune responses. MSCs display a reduced proliferation capacity and a high grade of apoptosis and senescence, and consequently their immunosuppressive and anti‐inflammatory activities are strongly compromised.

## 2 Deregulated miRNAs in ITP: a molecular landscape

miRNAs function is to silence gene expression by binding to partially complementary sequences in the 3’ untranslated region of target transcripts, leading to mRNA degradation or inhibition of translation (Zhao et al., 2022; O'Brien et al., 2018). miRNA genes are first transcribed into a primary transcript, known as pri-miRNA, and then cleaved by a microprocessor complex consisting of DiGeorge syndrome critical region gene 8 (DGCR8) and Drosha into a ∼70-nt stem-loop precursor, called pre-miRNA, within the nucleus (Bofill-De Ros and Vang Ørom, 2024). Next, the pre-miRNA moves to the cytoplasm via the Exportin 5 (XPO5)/RanGTP complex, where it is processed by the RNase III endonuclease Dicer into a mature ∼21-nt miRNA duplex (Bofill-De Ros and Vang Ørom, 2024; Kim et al., 2016). Only one of the strands of the duplex is chosen to be the guide and loaded into Argonaute proteins (AGOs) to assemble the RNA-induced silencing complex (RISC), which orchestrates the whole gene silencing machinery (O'Brien et al., 2018; Bofill-De Ros and Vang Ørom, 2024). Emerging evidence suggests that miRNAs are indispensable for maintaining immune system balance, thus when this regulatory machinery fails, miRNAs drive the pathogenesis of various autoimmune diseases, including ITP (Artimovič et al., 2024; Gaál, 2024). Altered miRNA expression disrupts immune tolerance, referring to the ability of the immune system to distinguish between the self and the non-self, lymphocyte subtype differentiation and platelet production (Zhao et al., 2022). Table 1 summaries some miRNAs which, according to reports, are deregulated in pediatric ITP, highlighting the available evidence regarding their association with the pathogenesis of the disease and their functional contribution to it.

**TABLE 1:** An overview of the various deregulated miRNAs in pediatric ITP**.** The table summarizes some of the miRNAs found to be deregulated in pediatric ITP. The altered expression of these miRNAs, in turn, leads to deregulation of their respective targets and pathways. Their role in contributing to the pathogenesis of ITP is described.

miRNA	Expression in pediatric ITP	Target/Pathway	Context	Clinical/Translational evidence	Pathogenic role	Ref.
miR-155	Upregulated	SOCS1	Cell-associated (PBMCs/macrophages)	Clinical association + mechanistic evidence	Negative correlation with platelet count and IL-4, IL-10, TGF-β1; positive correlation with IL-17A; M2 polarization suppression through the SOCS1-dependent PD1/PDL1 pathway	(Chang et al., 2020; Qian et al., 2014)
miR-146a	Downregulated in newly diagnosed and chronic ITP; Higher in chronic vs. newly diagnosed	IRAK1-TRAF6/NF-κB	Cell-associated (PBMCs); EV-associated (BMSC-derived EVs)	Clinical association + mechanistic evidence	Positive correlation with Treg cells and platelet count; negative correlation with Th17 frequency	(He et al., 2021; Gilyazova et al., 2023; Liu et al., 2016; El Zaiat et al., 2025)
miR-142-3p	Downregulated in adult ITP PBMCs; increased expression reported by Qian et al.	Not directly established in ITP	Cell-associated (PBMCs)	Clinical association	Negative correlation with Th17 cells; regulation of Treg suppressive function	(Liu et al., 2016; Huang et al., 2023; Anandagoda et al., 2019; Qian et al., 2018)
miR-223-3p	Deregulated	Not directly established in ITP	Circulating; platelet-derived	Clinical association	Not yet established	(Zhao et al., 2022; Charlon-Gay et al., 2026; Leng et al., 2022; Dang and Leelahavanichkul, 2020; Zhang et al., 2025)
miR-98-5p	Upregulated in adult ITP	IGF2BP1/IGF-2/PI3K-Akt; β-TrCP-p53	Cell-associated (adult ITP-MSCs)	Clinical association + mechanistic evidence	Upregolation of miR-98-5p promotes MSC apoptosis	(Wang et al., 2020)
miR-199a-5p	Downregulated in ITP mouse model	STAT3	EV-associated (engineered ADSC-derived EVs); tissue/cell-associated in ITP mice (spleen/CD4+ T cells)	Preclinical therapeutic evidence: administration of miR-199a-5p agomiR or miR-199a-5p-enriched ADSC-EVs inhibits pro-inflammatory Th17 differentiation and rescues platelet counts in ITP mouse model	Downregulation of miR-199a-5p promotes pro-inflammatory Th17 cell differentiation and disrupts the Th17/Treg balance, leading to immune-mediated platelet destruction	(Zhao et al., 2022; Li et al., 2021)
miR-641	Upregulated in adult ITP	SATB1/STIM1	Cell-associated (T cells)	Clinical association + preclinical therapeutic evidence: inhibition of miR-641 restores the Th17/Treg balance in ITP models	Th17/Treg imbalance	(Zhu et al., 2024)
miR-557	Upregulated	Akt/ERK pathway	Circulating; cell-associated (megakaryocytes)	Clinical association + preclinical therapeutic evidence: inhibition of miR-557 increases the proportion of Treg cells and the numbers of platelets and megakaryocytes in ITP mouse model	Negatively regulates TPO-induced megakaryocyte differentiation	(Wang et al., 2021)
miR-99a	Downregulated in adult ITP	mTOR	Cell-associated (CD4^+^ T cells)	Clinical association + mechanistic evidence	Th17/Treg imbalance	(Hua et al., 2019)
miR-125-5p	Downregulated	CXCL13	Cell-associated (PBMCs)	Clinical association + mechanistic evidence	Negative correlation with CXCL13 expression, contributing to immune dysregulation in ITP	(Li et al., 2015)

### 2.1 miR-155: the central regulator of inflammation

Despite the well-documented evidence of miR-155 as an oncomiR in T-cell lymphomas and various solid tumors, it also acts as a key factor in immune and inflammatory dysfunction in ITP (Chang et al., 2020; Mahesh and Biswas, 2019; Qian et al., 2014). miR-155 is expressed in haematopoietic stem and progenitor cells (HSPCs) and in mature haematopoietic cells, which include monocytes, granulocytes, B cells and T cells, and acts as a key regulator of immune cell responses (Alivernini et al., 2017). miR-155 contributes to T cell activation and differentiation and to the regulation of the Treg/Th17 balance, thus influencing pro-inflammatory immune responses. It regulates B cell proliferation and activation, germinal center formation, and antibody production (Alivernini et al., 2017). Its expression is upregulated in peripheral blood mononuclear cells (PBMCs) of adult ITP patients and is inversely correlated with both platelet count and anti-inflammatory mediators, including cytokine signaling suppressor 1 (SOCS1) mRNA, interleukin (IL)-4, IL-10, and transforming growth factor β1 (TGF-β1). On the contrary, it is positively correlated with plasma levels of the pro-inflammatory cytokine IL-17A (Qian et al., 2014). Recent studies focusing specifically on pediatric ITP have also reported increased expression of miR-155 in PBMCs from pediatric patients with newly diagnosed ITP compared with healthy controls, with higher expression observed in patients with severe disease. In this pediatric cohort, miR-155 expression was positively associated with the pro-inflammatory T helper 17 (Th17)/anti-inflammatory regulatory T (Treg) ratio and negatively associated with Treg cells and platelet count, suggesting an association between miR-155 expression and an imbalance in T cell subsets in pediatric ITP (Wang Z. et al., 2026). In the context of ITP, the available evidence mainly concerns cell-associated miR-155 expression, while its potential association with EVs and EV-mediated transfer remains to be clarified. In other immune contexts, miR-155 has been shown to modulate Th17/Treg balance and Th17 cell function through targeting SOCS1, supporting a potential role of this pathway in inflammatory immune dysregulation (Alivernini et al., 2017). In other inflammatory contexts, miR-155 hyperactivates the JAK-STAT (JAnus Kinase-Signal Transducers and Activators of Transcription) signaling pathways by directly suppressing negative regulators such as SOCS1, thereby blocking the resolution of inflammatory cascades (Bidgood et al., 2024; Wang et al., 2018). Consequently, targeting this axis represents a promising therapeutic strategy. Consistent with these findings, it has been demonstrated that in PBMCs and macrophages from ITP patients, miR-155-5p was upregulated whereas SOCS1 was downregulated. *In vitro* gain- and loss-of-function experiments showed that inhibition of miR-155-5p or upregulation of SOCS1 promoted polarization of macrophages towards the M2 phenotype, as indicated by an increased M2/M1 ratio, through the SOCS1-dependent Programmed cell death protein 1 (PD1)/Programmed cell death ligand 1 (PDL1) pathway (Chang et al., 2020). Overall, clinical evidence in pediatric ITP supports an association between increased miR-155 expression, inflammatory T cell imbalance, and disease severity, whilst the mechanistic evidence regarding the miR-155/SOCS1 axis still derives largely from studies conducted in adults and *in vitro*. Further functional studies in pediatric ITP are needed to determine whether these mechanisms are conserved in pediatric patients and whether they have therapeutic relevance.

### 2.2 miR-146a: the inflammation brake

miR-146a serves as a critical negative regulator of inflammatory responses, limiting excessive signalling by the innate immune system and maintaining immune homeostasis. Its anti-inflammatory function is primarily mediated through the repression of key signaling molecules involved in nuclear factor kappa-light-chain-enhancer of activated B cells (NF-κB) activation, thereby providing a negative-feedback mechanism that prevents protracted inflammatory responses (Gilyazova et al., 2023). miR-146a targets and represses two specific targets: TNF receptor-associated factor 6 (TRAF6) and IL-1 receptor-associated kinase 1 (IRAK1), two signal transducers upstream of NF-κB (Gilyazova et al., 2023). Following stimulation of cell surface receptors, like the interleukin-1 receptor (IL-1R) or members of the Toll-like receptor (TLR) family, downstream signaling is activated via the MyD88-dependent pathway, which recruits IRAK1 and TRAF6 (Pe et al., 2023). They trigger a cascade that leads to the activation and nuclear translocation of NF-κB, where it induces the transcription of various target genes, including pro-inflammatory chemokines IL-8, C-X-C motif chemokine ligand 1 (CXCL1), and Chemokine C-C motif ligand 5 (CCL5), as well as the primary transcript pri-miR-146a (Gilyazova et al., 2023; Rusca and Monticelli, 2011). A negative feedback loop is established, where the mature miR-146a suppresses IRAK1 and TRAF6 expression. The downregulation of these two targets attenuates the subsequent NF-κB-mediated cytokine production, thereby preventing inflammatory hyperactivation. In a non-pediatric ITP cohort, miR-146a expression was downregulated and positively correlated with Treg cell frequency and platelet count and negatively correlated with Th17 cell frequency (Liu et al., 2016). In pediatric patients with ITP reduced plasma miR-146a-5p levels were reported in both newly diagnosed and chronic ITP compared with healthy controls (El Zaiat et al., 2025). These findings concern circulating miR-146a-5p and do not, in themselves, establish its association with EVs. EVs derived from bone marrow MSCs (BMSCs) offer a method for delivering functional miR-146a-5p to target CD4^+^ T cells, helping to correct the Th17/Treg imbalance observed in ITP through the direct targeting and repression of endogenous IRAK1 (He et al., 2021). The restoration of the miR-146a-5p/IRAK1 pathway mediated by BMSC EVs thus provides a viable cell-free therapeutic approach for clinical intervention (Figure 2). In addition to this link to the Th17/Treg axis, miR-146a-5p may also contribute to the Th1/Th2 balance. In pregnant patients with ITP, reduced levels of miR-146a-5p have been associated with increased expression of Caspase recruitment domain family member 10 (CARD10), while miR-146a-5p derived from BMSC-EVs was shown to modulate the Th1/Th2 balance via the CARD10/NF-κB pathway. Consistent with this mechanism, it has been demonstrated *in vitro* that CARD10 suppresses Th2 polarization and enhances Th1 responses, potentially contributing to the pro-inflammatory environment associated with ITP (Liu et al., 2026; Rong et al., 2024). Therefore, while clinical studies support an association between reduced miR-146a-5p expression and ITP, including pediatric ITP, the proposed mechanisms involving IRAK1 and CARD10 remain largely supported by experimental evidence.

**FIGURE 2:**
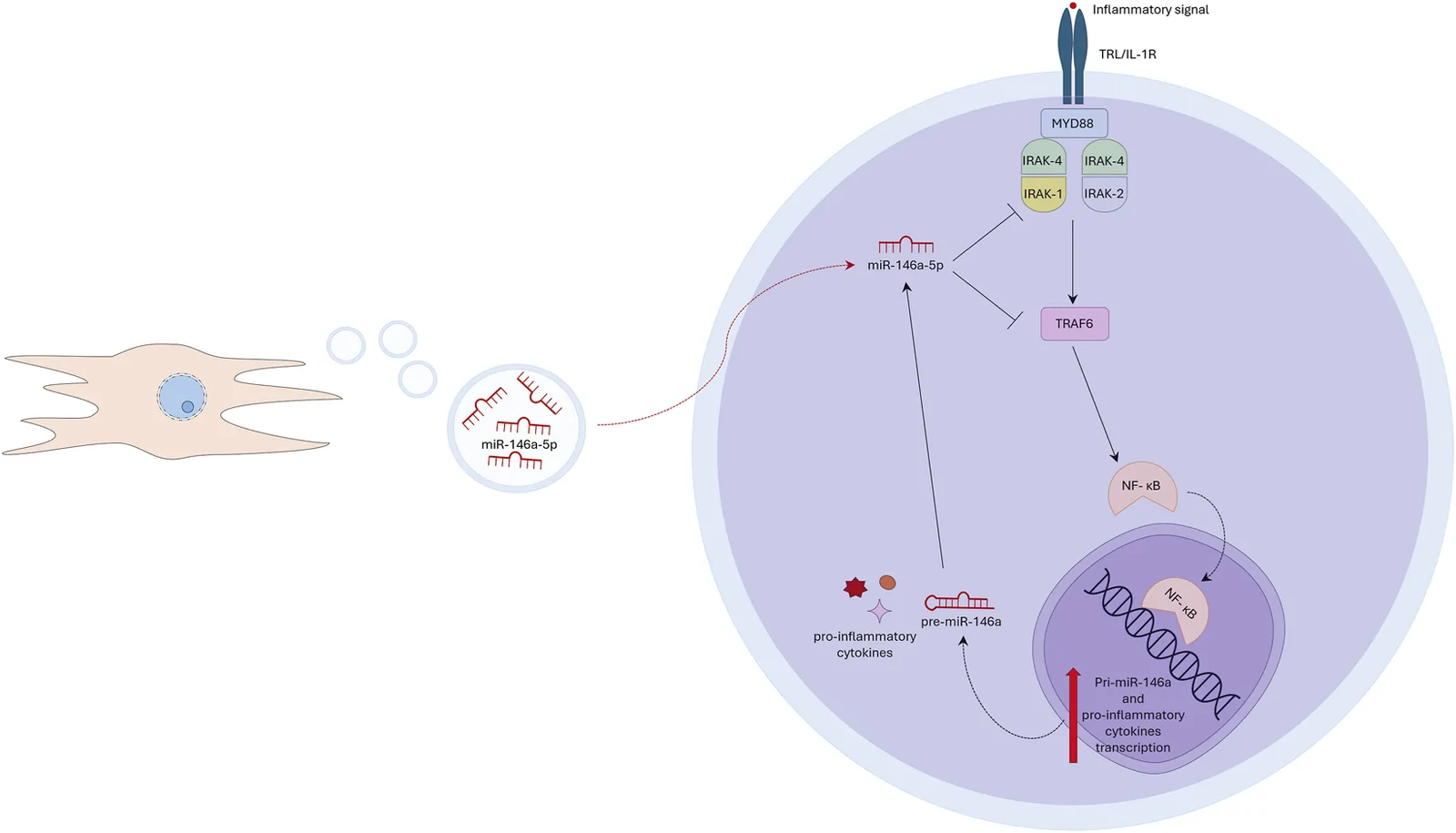
miR-146a-5p negative regulation of IRAK-1/TRAF6/NF-κB pathway. Following stimulation of cell surface receptors, like the IL-1R or members of TLR family, downstream signaling cascade leads to the activation and nuclear translocation of NF-κB, where it induces the transcription of various targets, including pro-inflammatory chemokines and pri-miR-146a. A negative feedback loop is established, where the mature miR-146a suppresses IRAK1 and TRAF6 expression and contributes to attenuate the subsequent NF-κB-mediated inflammatory activation.

### 2.3 miR-142: a role in immune tolerance

miR-142 is expressed preferentially among haematopoietic cells, playing a key role in haematopoietic homeostasis (Huang et al., 2023). miR-142 contributes to the regulation of immune-cell activation, differentiation, and homeostasis (Olson and Derudder, 2023; Sharma, 2017). The expression of miR-142 is found to be deregulated in autoimmune diseases and hematological malignancies, such as leukemia and lymphoma (Huang et al., 2023). miR-142 knockout mice model displays a significant reduction in Treg cells in both thymus and secondary lymphoid organs, suggesting that it plays a key role in Treg cells development and stability (Wang et al., 2022). In Treg cells, miR-142-5p is the predominant isoform, whose target is cAMP-hydrolyzing enzyme phosphodiesterase-3b (PDE3B) mRNA (Anandagoda et al., 2019). PDE3B hydrolyzes its substrates cAMP, accelerating intracellular cAMP turnover. As the immunosuppressive function of Treg cells depends on a high cAMP intracellular concentration, PDE3B inhibition is required for the maintaining of these elevated levels (Anandagoda et al., 2019). Mechanistic studies have demonstrated that the specific deletion of miR-142 in Treg cells increased PDE3B expression, resulting in a decreased intracellular cAMP level and impaired suppressive function of Treg cells, whilst inhibition or genetic ablation of PDE3B restored the suppressive activity of Treg cells (Anandagoda et al., 2019). These observations suggest that the miR-142/PDE3B/cAMP axis participates in the maintenance of Treg cell function and peripheral immune tolerance. miR-142-3p is generally reported to be downregulated in adult ITP patients and shows a significant negative correlation with the frequency of Th17 cells (Liu et al., 2016). However, it was found to be upregulated in PBMCs of the ITP patient cohort examined by Qian C et al., probably due to differences in patient demographics, geographic origin, disease status, and the limited sample size of the study (Qian et al., 2018). Although the miR-142-5p/PDE3B/cAMP pathway provides a mechanistic basis for its role in Treg function, this mechanism has not been directly demonstrated in pediatric ITP. Overall, the resulting Th17/Treg imbalance promotes the persistent autoimmune response that causes increased ⁠platelet destruction.

## 3 EVs selective miRNA loading

For many years, EVs were considered cellular waste and received little attention. However, the development of standardized isolation and detection methods led to a rapid expansion of EV research (Aloi et al., 2024). EVs are a heterogeneous population of particles delimited by a lipid bilayer and released by diverse cell types, such as immunoregulatory cells, including MSCs, Treg cells, and dendritic cells, or tumor cells or tumor cells (Buzas, 2023; Kumar et al., 2024; Shah et al., 2025; Welsh et al., 2024). They are unable to replicate due to the absence of functional nuclear material and their production is often enhanced by specific signals, such as cell activation, oxidative stress, hypoxia, and other pathological conditions (Welsh et al., 2024; Żmigrodzka et al., 2016). EVs mediate intercellular communication and signaling by transporting a wide range of bioactive molecules, such as proteins, lipids, and nucleic acids to recipient cells and thereby modulating immune responses (Aloi et al., 2024; Kumar et al., 2024; Shah et al., 2025). Immunoregulatory cell-derived EVs can transfer the functional properties of their parent cells to recipient cells and influence the differentiation of immune cells, cytokine production and other aspects of the immune microenvironment, confirming their potential as cell-free immunomodulatory therapies in autoimmune diseases (Shah et al., 2025). EVs secreted under pathological conditions reflect the pathological states of their originating cells, and these profiles represent innovative, non-invasive biomarkers for early detection, prognosis and disease progression across a variety of conditions (Xu et al., 2022). In autoimmune diseases, these EVs act as messengers that can alter inflammatory responses (Chen et al., 2026; Duan et al., 2019).

EVs can be classified into small EVs (<200 nm in diameter) and large EVs (>200 nm) (Welsh et al., 2024; Lee et al., 2023). It is important to note this kind of classification may include mixed populations, as separation methods do not yield perfectly homogeneous vesicle subsets. They can be also classified by biogenesis into exosomes, ectosomes (also referred to as microvesicles or microparticles) and apoptotic bodies (Welsh et al., 2024). Exosomes (0.03–0.15 μm) originate from the endosomal system and are generated through the fusion of multivesicular bodies with the plasma membrane (Li et al., 2026). Ectosomes (0.1–1 μm) refer to EVs from the cell surface, generated by the outward budding of the plasma membrane during cell activation (Surman et al., 2017). Apoptotic bodies are larger vesicles (1–5 μm) released during programmed cell death and may contain nuclear and cellular fragments (Shi et al., 2025). Alternatively, EVs are also classified using their cellular origin, including platelet microparticles (PMPs), erythrocyte microparticles (RBC-MPs), endothelial cell microparticles (EMPs), and tumor cell microparticles (TMPs). Oncosomes are another type of EVs, representing large vesicles (1–10 μm) released by migrating tumor cells (Żmigrodzka et al., 2016) (Figure 3).

**FIGURE 3:**
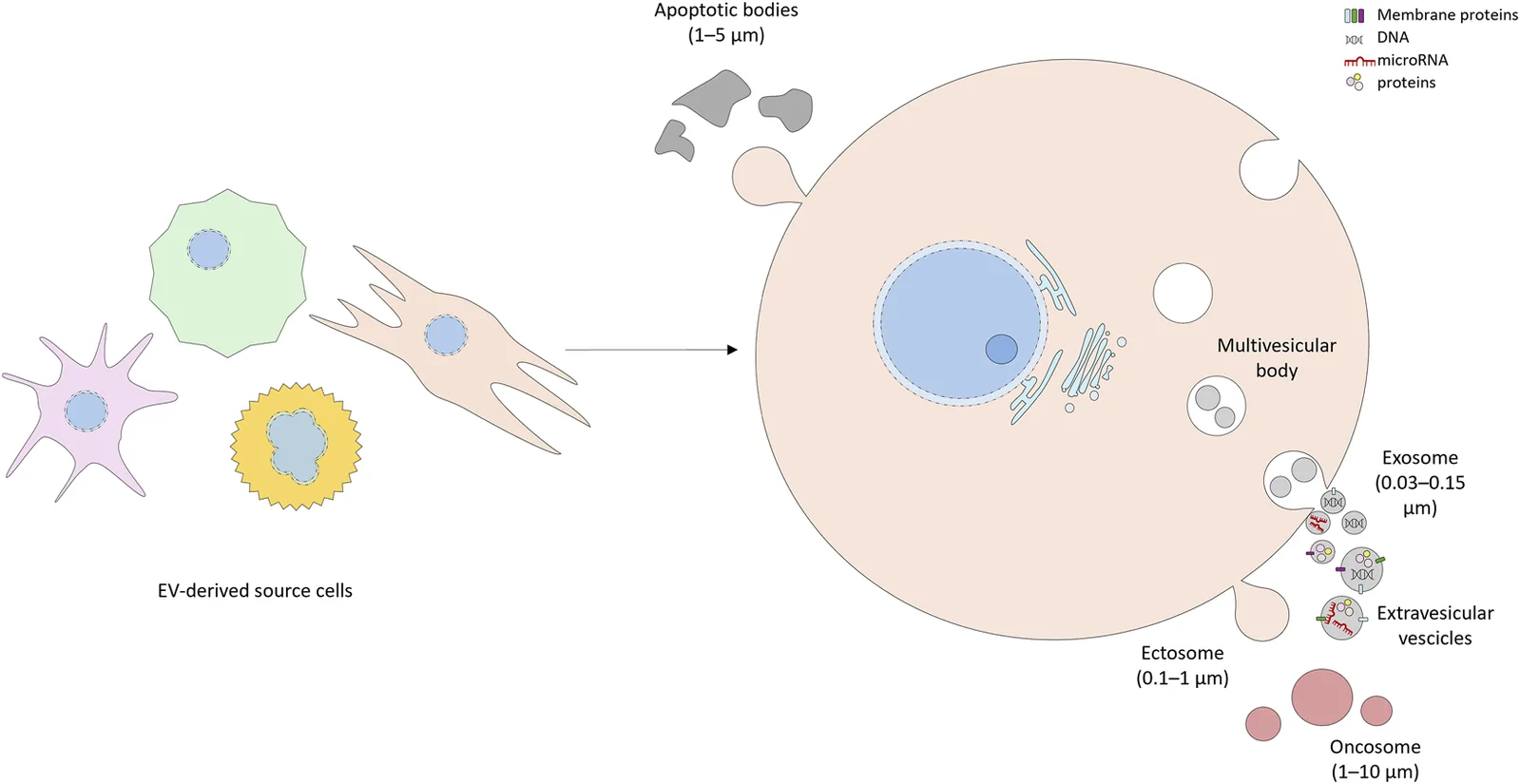
Biogenesis of extracellular vesicles. EVs are released by all cell types, including immune, stem, or tumor cells. Exosomes (0.03–0.15 μm) are secreted via the endosomal system, through the formation of multivesicular bodies, which fuse with the plasma membrane. In contrast, ectosomes (0.1–1 μm) originate directly from the plasma membrane, by budding from the plasma membrane. Apoptotic bodies (1–5 μm) and oncosomes (1–10 μm) are large vesicles, both released from membrane blebs of dying cells and tumor cells, respectively. EVs are particles delimited by a lipid bilayer, containing complex cargo, including membrane proteins and other proteins, DNA, non-coding RNAs, such as miRNAs.

Unfortunately, these size- and biogenesis-based definitions should be applied cautiously, as current isolation methods do not allow complete separation of EV subtypes with distinct origins, and no universal markers for ectosome, exosome or other EV subtypes exist. EV classification remains a subject of ongoing debate, due to their different sizes, cellular origins, and biogenesis pathways and the limitations of current isolation and characterization techniques. The International Society for Extracellular Vesicles (ISEV) in the “Minimal information for studies of extracellular vesicles 2018 (MISEV2018)” statement officially recognises this heterogeneous population of vesicles under the collective term of extracellular vesicles (Welsh et al., 2024). For the purposes of this review, EVs will be used in a broad sense, focusing mainly on their miRNA content.

EVs are enriched with noncoding RNAs, including miRNAs, tRNA, rRNA, mitochondrial RNA, circular RNA (circRNA), and long noncoding RNA (lncRNA) (Liu and Halushka, 2025). Several databases, such as Vesiclepedia, ExoCarta, and EVpedia, have been developed to catalogue EV cargo. More recently, EVmiRNA2.0 was developed as an expanded platform to provide for the comprehensive analysis of EV miRNA expression profiles and their potential roles in pathological diseases (Xie et al., 2026). Several distinct molecular pathways govern the sorting of miRNAs into EVs, which can be broadly categorized into RNA-binding proteins (RBP)-dependent and RBP-independent mechanisms (Huang et al., 2025). RBPs, including AGO2 and heterogeneous nuclear ribonucleoproteins (hnRNPs), act as shuttles capable of targeting specific miRNAs to control the loading into EVs for extracellular transport (Liu and Halushka, 2025; Groot and Lee, 2020; Marocco et al., 2025; O'Grady et al., 2022). HnRNPs recognize and bind unique sequence motifs within the miRNAs, known as EXOmotifs, such as GGAG/UGCA, driving their sequestration into EVs (Huang et al., 2025; Villarroya-Beltri et al., 2013). Recent findings indicated that AGO2-miRNAs represent the majority of free human plasma miRNAs, suggesting that AGO2 might have an important role in stably binding and sorting miRNAs into EVs (Groot and Lee, 2020). By contrast, RBP-independent sorting mechanisms have been associated with lipid-based microdomains, such as cholesterol and sphingolipid-enriched membrane regions, which serve as platforms for the passive EV incorporation of miRNAs (Huang et al., 2025). The encapsulation of miRNAs within EVs contributes to their stability and prevents degradation by extracellular RNases (Yáñez-Mó et al., 2015). Upon reaching their destination, EV-miRNAs can then be taken up by recipient cells through various mechanisms, including endocytosis, phagocytosis, or direct fusion with the plasma membrane, depending on the cell type and the surrounding microenvironment (Guo et al., 2024). EV-miRNAs modulate essential processes such as proliferation, differentiation, apoptosis, and immune responses, contributing to both physiological homeostasis and the development of various diseases, due to their role in intercellular communication (Wu et al., 2026). Despite their therapeutic potential, the clinical application of EV-based therapies continues to be challenged by several difficulties, most notably the standardization of EV isolation and profiling, targeting efficiency, and the lack of a complete assessment of long-term safety (Shah et al., 2025).

### 3.1 Platelet microvesicles as miRNA carriers in ITP

Platelet-derived extracellular vesicles (PDEVs) constitute a heterogeneous population of vesicles released by platelets and their precursor cells, the megakaryocytes. Platelet-derived microvesicles, also known as platelet microparticles (PMPs), are generated predominantly through outward budding and detachment from the plasma membrane of activated platelets, whereas megakaryocytes can also release extracellular vesicles directly (Stone et al., 2022). PMPs represent a major population of circulating extracellular vesicles, characterized by a heterogeneous EV population ranging from 0.1–1 μm. Following platelet activation, they are produced and released from the cellular plasma membrane (Jolink et al., 2021). They retain platelet-derived membrane proteins and receptors and carry a diverse cargo of lipids, proteins, enzymes, and nucleic acids, including mRNAs and miRNAs, which can be transferred to recipient cells (Puhm et al., 2021). PMPs can influence the functions of recipient cells through these interactions, which are mediated by the substances they carry and are involved in intercellular communication, inflammatory responses and immune regulation (Puhm et al., 2021). In addition to their role in thrombosis and thrombopoiesis, PMPs may also be involved in immune regulation by interacting with macrophages and other immune cells. Recent experimental evidence indicates that platelet-derived EVs can modulate macrophage responses, with immunomodulatory effects that depend on the activation state of the parent platelets. Moreover, PMPs may contribute to the communication between platelets and immune cells and potentially influence adaptive immune responses through the transfer of bioactive molecules (Palviainen et al., 2024). However, these immunomodulatory effects have been mainly investigated in non-ITP contexts, and their specific contribution to the immune dysregulation of ITP remains to be established. Unlike the platelets, PMPs are able to easily enter the lymphatic system and bone marrow, where they can act as crucial epigenetic signals by transferring specific transcripts (Nelson et al., 2021). miR-1915-3p is highly enriched in PMPs and is transferred to HSPCs, where it directly represses the B member of the Rho GTPase family (RHOB), promoting megakaryocytic differentiation and platelet production. *In vitro* uptake of PMPs by HSPCs and the overexpression of miR-1915-3p have validated the miR-1915-3p/RHOB regulatory axis, while the administration of PMPs increased the number of megakaryocytes and platelets *in vivo* (Qu et al., 2020). It remains to be determined whether this PMP-mediated miR-1915-3p/RHOB pathway is altered in the pathological context of ITP. However, an altered PMP burden could potentially contribute to the dysregulation of thrombopoiesis and immune homeostasis within the bone marrow niche in ITP (Nelson et al., 2021). In many studies, miR-223-3p was described as one of the most abundant platelet-derived miRNAs (Charlon-Gay et al., 2026; Leng et al., 2022). miR-223 is a critical haematopoietic miRNA that serves as a master regulator of immune cell development, inflammation, predominantly enhancing megakaryocyte differentiation and maturation by inhibiting Myosin Heavy Chain 10 (MYH10) and LIM Domain Only 2 (LMO2) (Leng et al., 2022; Jiao et al., 2021). miR-223 also exerts its anti-inflammatory effects, maintaining homeostatic macrophage polarization. Experimental studies have shown that inhibition of miR-223 expression alters macrophage polarization, whereas restoration of its expression promotes anti-inflammatory M2 transition. Its targets are key regulatory metabolic pathways, including the transcription factor Hypoxia-inducible factor 1-alpha (HIF-1α), Signal transducer and activator of transcription 3 (STAT3), and the Forkhead box protein O1 (FOXO1)/Peroxisome proliferator-activated receptor gamma (PPARγ) signaling axes (Dang and Leelahavanichkul, 2020; Zhang et al., 2025). In ITP, an imbalance of M1/M2 ratio has been observed, with a prevalence of M1 macrophages, responsible for an increased release of pro-inflammatory cytokines, such as IL-6, TNF-α and Interferon-γ (IFN-γ), driving the underlying immune deficiency (Di Paola et al., 2020). Notably, altered miR-223-3p expression has also been reported in the plasma of pediatric patients with ITP, supporting its potential involvement in the disease context (Bay et al., 2014). However, despite its established roles in megakaryocyte and macrophage biology, the specific pathogenic contribution of this miRNA to ITP has not yet been established. Future research will need to isolate and specifically map the miRNA cargo contained within PMPs, as miRNAs are mostly measured as free circulating miRNAs in plasma and not specifically measured within PMPs. The evaluation of EV-miRNAs depends on pre-analytical conditions and EV isolation procedures. Residual platelets may co-isolate with EV-enriched plasma fractions and release additional platelet-derived miRNAs during sample processing, potentially confounding the quantification of EV-miRNAs. Indeed, platelet removal has been shown to improve the detection and reproducibility of EV-miRNA measurements. Therefore, standardized blood collection and processing, adequate control of platelet contamination, and EV characterization according to current MISEV recommendations are essential for the reliable evaluation of EV-miRNAs as biomarkers (Welsh et al., 2024; Bettin et al., 2022; Bracht et al., 2023). This targeted molecular understanding will pave the way for new diagnostic and therapeutic strategies for ITP.

### 3.2 Immune cell-derived EVs as miRNA Carriers in ITP

EVs produced by immune cells represent a crucial field of miRNA-mediated intercellular communication, which remains largely unexplored in ITP. Immune cell-derived EVs exhibit immunomodulatory effects ranging from interactions between immune cells to the balance of T helper subset and the polarization of macrophages (Wu et al., 2026). EVs could provide promising therapeutic platforms for the delivery of functional miRNAs, thanks to their high biocompatibility, low immunogenicity, intrinsic ability to cross biological barriers, and the possibility of being engineered for targeted delivery to specific cells or tissues (Liu et al., 2024; Luisotti et al., 2025). Among the various sources of EVs, those derived from MSCs have attracted particular interest due to their intrinsic regenerative and immunomodulatory properties and are currently being studied as cell-free therapeutic alternatives for both oncological and non-oncological diseases, including autoimmune diseases (Rajendran and Gangadaran, 2026; Zhou and Shan, 2026). MSCs are a heterogeneous cell type that can be isolated from a variety of various tissues, such as adult adipose tissue, peripheral blood, endometrial polyps, menstrual blood, bone marrow, neonatal placenta, and umbilical cord tissues, able to proliferate extensively and to differentiate into different cell types such as osteoblasts, adipocytes, and chondrocytes (Clark et al., 2014; Xu et al., 2024). MSCs exert their anti‐inflammatory and immunomodulatory functions by actively interacting with components of the innate immune system through secreting EVs, mainly showing anti-inflammatory effects (Luque-Campos et al., 2021). As previously stated, MSCs from ITP patients (ITP-MSCs) are functionally defective, displaying proliferation defects, increased apoptosis, and loss of immunoregulatory function, with an increased pro-inflammatory cytokine profile, which concur in ITP pathogenesis (Di Paola et al., 2020; He et al., 2021). Nowadays, miRNAs have emerged as pivotal regulators of MSCs proliferation, differentiation, and paracrine functions, previously described as impaired in ITP (Clark et al., 2014; Yang et al., 2025). miR-146a represents one of the most promising therapeutic candidates in ITP as it acts as a negative regulator of NF-κB signaling and is consistently downregulated in patients with ITP (He et al., 2021). The therapeutic potential of miR-146a-engineered EVs is supported by studies conducted on other inflammatory diseases, in which they effectively suppress the IRAK1/TRAF6/NF-κB signaling pathway and restore immune homeostasis, reinforcing their potential application in ITP (Yang et al., 2025; Shahryari et al., 2025) (Figure 2). Beyond miR-146a-5p, other MSC-derived miRNAs involved in ITP pathogenesis have been described. miR-98-5p represents an important pathogenic hub because it is increased in ITP-MSCs and promotes cell apoptosis by targeting insulin-like growth factor 2 mRNA-binding protein 1 (IGF2BP1), leading to the downregulation of its downstream target, insulin-like growth factor 2 (IGF-2) (Wang et al., 2020). Experimental modulation of miR-98-5p in ITP-MSCs confirmed its functional role: miR-98-5p overexpression increased MSC apoptosis, whereas its inhibition reduced apoptosis and restored IGF2BP1/IGF-2 (Liu et al., 2024). The subsequent downregulation of IGF-2 causes inhibition of the phosphatidylinositol 3-kinase (PI3K)/Akt pathway, which is involved in the process of MSC deficiency (Li et al., 2021). Additionally, miR-98-5p upregulates p53 by inhibiting β-transducin repeat-containing protein (β-TrCP)-dependent p53 ubiquitination. Importantly, the MSCs investigated in this study were derived from adult patients with ITP, and the relevance of the miR-98-5p/IGF2BP1/IGF-2 axis to pediatric ITP remains to be established. A further point of interest is the role of MSCs in promoting alternative regulatory functions, such as the induction of regulatory B cells, a subset of B cells (Chen et al., 2021). In this context, umbilical cord-derived MSCs (UC-MSCs) can promote regulatory B cells differentiation via miR-133-dependent EVs. Taken together, these findings suggest that MSCs should not be classified merely as dysfunctional targets within the ITP microenvironment, but rather as a source of therapeutic EVs capable of bringing about comprehensive immune remodulation (Figure 4).

**FIGURE 4:**
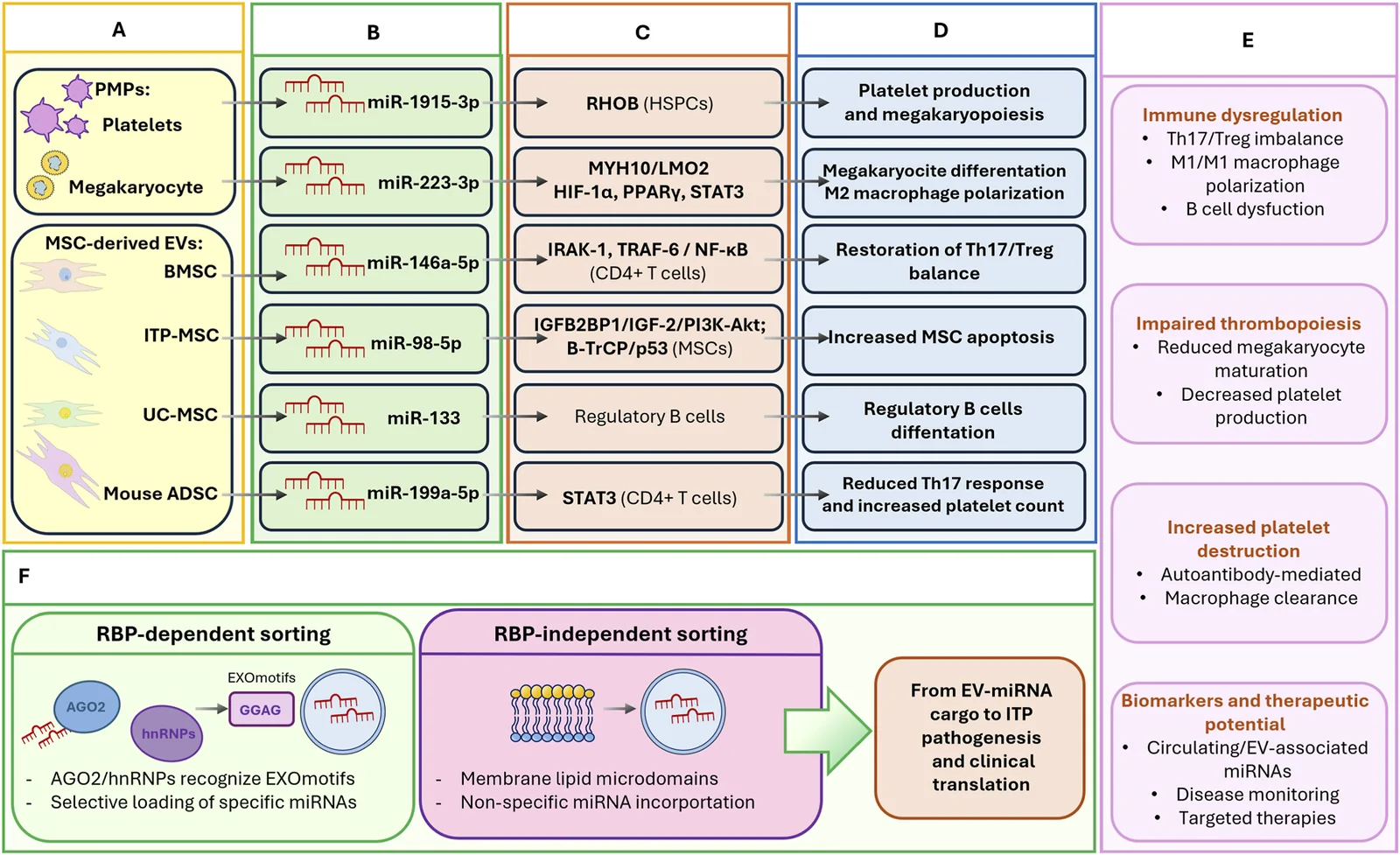
Integrative overview of extracellular vesicle-associated miRNAs and their potential roles in ITP. EVs released by platelets/megakaryocytes and mesenchymal stromal cells (MSCs) can transport specific miRNAs to recipient cells, thereby modulating molecular pathways involved in thrombopoiesis, immune-cell differentiation, inflammation, and immune homeostasis. Platelet-derived microvesicles can transfer miR-1915-3p to haematopoietic stem/progenitor cells (HSPCs), promoting megakaryocytic differentiation and platelet production through the RHOB pathway, while platelet-derived miR-223-3p contributes to megakaryocyte maturation and macrophage polarization. MSC-derived EVs carrying miR-146a-5p can modulate the IRAK1/NF-κB pathway and restore the Th17/Treg balance, whereas miR-199a-5p-enriched MSC-derived EVs have been associated with inhibition of Th17 differentiation. Other MSC-associated miRNAs, including miR-98-5p and miR-133, have been implicated in MSC apoptosis and regulatory B-cell differentiation, respectively. miRNA sorting into EVs may occur through RNA-binding protein-dependent mechanisms, involving proteins such as AGO2 and hnRNPs, or through RNA-binding protein-independent mechanisms involving lipid-enriched membrane domains. **(A)** Cell of origin **(B)** EV-miRNA **(C)** Target cell / pathway **(D)** Functional effect **(E)** Impact on ITP pathogenesis **(F)** Mechanisms of miRNA sorting into EVs.

## 4 AntagomiRs and miRNA mimics: new therapeutic frontiers in ITP

Recent advances in miRNA-targeted strategies have highlighted the role of the epigenetic profile in ITP, including DNA methylation, histone modifications, and non-coding RNA, and their potential as new therapeutic targets capable of influencing the course of the disease (Tan et al., 2023). miRNAs discussed in this section were selected based on the availability of experimental evidence supporting their therapeutic modulation using antagomiRs, miRNA mimics, or related miRNA-targeting approaches in ITP. AntagomiRs or miRNA mimics offer a promising targeted strategy for manipulating miRNA expression, aimed at restoring cellular homeostasis. AntagomiRs are small, chemically modified miRNA antagonists that act as direct or functional structural inhibitors, perfectly complementing the specific miRNA target (Guo et al., 2023; Lima et al., 2018). These function as artificial inhibitors of target miRNAs, by transiently repressing their expression. Following binding to the target miRNA, antagomiR causes steric hindrance, preventing the miRNA from entering the RISC complex (Tan et al., 2023). This, in turn, leads to the derepression of the target transcripts, restoring normal translation and proper cellular function. Various strategies have been developed for the delivery of antagomiRs, including the use of nanoparticles, cationic polymers, transfection or microinjection techniques, but EVs have attracted growing interest as a delivery platform due to their high biocompatibility, and their potential to facilitate the transfer of therapeutic cargo to recipient cells (Luisotti et al., 2025; Guo et al., 2023). However, the therapeutic application of EV-miRNA delivery still faces important challenges, including efficient and specific targeting of recipient cells, optimization of miRNA loading and release, and control of cargo stability. In addition, the ability of miRNAs to regulate multiple target transcripts raises concerns regarding potential off-target effects and inadvertent modulation of biological pathways (Luisotti et al., 2025; Seyhan, 2024). In contrast to antagomiRs, there are inhibitors that enable long-term miRNA inhibition. This is the case of miRNA sponge, a specially designed RNA transcript expressed by DNA vectors that competitively binds to the target miRNA (Avendaño-Portugal et al., 2025). Once transfected and stably expressed in cells, the miRNA sponge can exert prolonged or permanent suppression against a single or multiple target miRNAs. A promising therapeutic approach for ITP is miR-155 targeting, considering its overexpression and its central role in promoting Th1/Th17 response and inhibiting Treg cells in ITP (Chang et al., 2020; Qian et al., 2014; Yao et al., 2012). Thus, a specific inhibitor of this specific miRNA could represent a potential novel application to target its pathogenic role. This hypothesis is confirmed by the development of Cobomarsen (MRG-106), an oligonucleotide inhibitor already tested in human clinical trials for some onco-hematological diseases, such as lymphomas and leukemias (Seto et al., 2018). miR-641 upregulation in peripheral T cells of adult ITP patients is associated with Th17/Treg imbalance, because miRNA targets and inhibits the expression of Special AT-rich sequence-binding protein 1 (SATB1) and Stromal interaction molecule 1 (STIM1), two important regulators of T cell differentiation and immune homeostasis (Zhu et al., 2024). In experimental models, both miR-641 sponge in ITP primary T cells *in vitro*, or antagomiR-641 injected *in vivo* in a murine ITP model caused the restoration of the expression of these two transcripts and of the Th17/Treg balance, modulation of the cytokine response, and improvement in platelet count, highlighting its potential as a therapeutic target. miR-557 is overexpressed in the plasma of ITP pediatric patients and negatively regulates the proliferation and maturation of TPO-induced megakaryocytes. Wang Y et al. study has demonstrated that inhibition of miR-557 using an antagomiR activates the protein kinase B (Akt)/Extracellular signal-regulated kinases (ERK) signaling pathway, resulting in reduction of proportion of CD4^+^ T lymphocytes in PBMCs and increases the proportion of Treg cells in CD4^+^ T lymphocytes in peripheral blood of ITP model rats (Wang et al., 2021). In conclusion, the use of antagomiRs represents an innovative frontier compared to traditional treatments for ITP, because these molecules target the specific molecular pathogenic target without causing generalized immunosuppression.

In addition to antagomiRs, miRNA mimics, a double-stranded synthetic RNA, are widely used in the study of miRNA characteristics and their contribution to the pathogenesis of various diseases. Since its function is to restore the endogenous function of a target miRNA, a miRNA mimic is employed when the miRNA expression is downregulated (Luisotti et al., 2025; Seyhan, 2024). To better understand the effect of miR-199a-5p on immune cells, in a study conducted by Li J et al., 2021, CD4^+^ T lymphocytes isolated from the spleen tissues of healthy mice were transfected with the miR-199a-5p agomiR, a chemically modified miRNA mimic that enhances its stability (Li et al., 2021). From a functional perspective, its overexpression blocks Th17 cell differentiation by acting directly on STAT3. Consistent with this mechanism, miR-199a-5p-enriched EVs derived from MSCs in the adipose tissue (ADSCs) of healthy mice inhibit pro-inflammatory Th17 cell differentiation and improve platelet counts in experimental models of ITP, supporting the therapeutic potential of EV-mediated administration of miR-199a-5p. Similarly, the upregulation of miR-146a expression in ITP patients with agomiR significantly reduces the differentiation of Th17 cells and elevates the differentiation of Tregs, thereby restoring immune homeostasis (Liu et al., 2016). The expression of miR-99a in CD4^+^ T lymphocytes from adult patients with ITP is reduced, and its overexpression, achieved via transfection with a miRNA mimic, increased Treg levels in patients with ITP by repressing mTOR (Hua et al., 2019). Studies conducted on pediatric patients with ITP have revealed elevated plasma levels of the CXC inflammatory chemokine ligand-13 (CXCL13). CXCL13 concentration decreases following therapeutic treatments, such as dexamethasone. The introduction of a miR-125-5p mimic into CD4^+^ T lymphocytes is capable of acting directly on the expression of CXCL13 and suppress it, mimicking the therapeutic effect of corticosteroids, whilst the miR-125-5p inhibitor has the opposite effect (Li et al., 2015). Although challenges regarding delivery and stability remain, loading these synthetic molecules into EVs could represent a potential cell-free therapeutic strategy for restoring immune tolerance. However, the available evidence in ITP remains largely preclinical, with most studies based on *in vitro* experiments or animal models. Further studies are needed to establish the long-term safety, biodistribution, therapeutic efficacy, and optimal dosing of these approaches before their translation into clinical practice, particularly in pediatric ITP.

## 5 miRNAs in EVs as non-invasive biomarkers in pediatric ITP

In ITP, where platelets, T and B lymphocytes, monocytes, and megakaryocytes all contribute to pathogenesis, this specificity becomes particularly valuable. The diagnosis of pediatric ITP remains one of exclusion and there are no validated diagnostic, prognostic or predictive biomarkers of therapeutic response (Allegra et al., 2023). The EV-miRNAs profile reflects the specificity of the tissue of origin, retaining the biological markers of the cell of origin (Barger et al., 2016). However, an active sorting mechanism selects and enriches specific miRNAs in EVs, transforming these vesicles from simple passive biomarkers to active targeted mediators of intercellular communication (Mori et al., 2019). A relevant methodological aspect concerns the choice between the analysis of free miRNAs and EV-associated miRNAs as biomarkers. In order to predict whether a newly diagnosed pediatric patient will go into spontaneous remission or chronic condition, circulating miR-146a-5p, miR-106b-5p and miR-200c-3p are promising ITP prognosis predictors (El Zaiat et al., 2025). The expression levels of miR-200c-3p and miR-146a-5p are higher in pediatric chronic ITP than in newly diagnosed ITP, whereas lower miR-106b-5p levels were associated with complete response to therapy (El Zaiat et al., 2025). It is important to highlight that these findings relate to circulating miRNAs and do not establish whether these molecules are specifically associated with EVs.

Several studies have shown that EV-miRNAs offer potential advantages over other types of biomarkers for several reasons, such as stability, since the encapsulation protects miRNAs from RNase degradation, and accessibility through a simple blood sample, making non-invasive monitoring possible (Xu et al., 2022; Arailym et al., 2026). Interestingly, AGO2-miRNAs represent the majority of free plasma miRNAs, but they are less specific to their originating cell than EV-miRNAs (Groot and Lee, 2020; Geekiyanage et al., 2020). Despite their potential, the use of EV-miRNAs as biomarkers faces significant challenges that require standardization. Firstly, EV isolation methods are still controversial: no universally accepted standard protocol for EV isolation, and different methods produce EV populations with varying compositions (Welsh et al., 2024). More generally, pre-analytical and methodological heterogeneity, including differences in sample type and processing, may contribute to variability in miRNA profiles across different ITP studies (Liu et al., 2026). Most current studies on miRNAs in ITP have been conducted on small cohorts. Multiple, multicenter, and prospective studies are needed to validate these potential biomarkers identified.

## 6 Conclusion

This review sheds light on the emerging interplay between miRNAs, EVs, and immune dysregulation in pediatric ITP. In this context, pediatric ITP shows unique biological and clinical characteristics, such as greater immunological plasticity and the ability to restore immune tolerance compared to adults, further emphasizing the importance of investigating EV-miRNAs. Notably, the immune system in childhood is characterized by dynamic changes in the Treg/Th17 balance, T cell maturation, and cytokine production, which are also reflected in miRNA expression profiles.

EVs derived from haematopoietic and immune cells, function as active mediators of intercellular communication by transporting functional miRNAs, which serve not only as biomarkers reflecting a particular cellular state, but as messengers capable of modulating biological processes. As the expression of several miRNAs, such as miR-155, miR-146a and miR-142, has been revealed to be altered in ITP and implicated in Treg/Th17 imbalance, increased inflammatory cytokine production and disrupted megakaryocytic differentiation, therapeutic targeting of their levels with antagomiRs or miRNA mimics could be an innovative approach for ITP treatment. The stability, accessibility, and tissue specificity of EV-miRNAs make them particularly advantageous as novel non-invasive diagnostic and prognostic biomarkers and therapeutic targets for ITP treatment.

Despite growing findings linking epigenetic mechanisms to ITP pathogenesis, EV-miRNAs profiles investigation in pediatric cohorts remain scarce. Most of the available mechanistic and therapeutic evidence originates from ITP in adults, from experimental animal models or from other inflammatory diseases and, therefore, may not be directly extrapolated to pediatric ITP. Future research should focus on large-scale, multicenter, prospective studies comparing EV-miRNAs with circulating free miRNAs in carefully characterized pediatric cohorts of patients with ITP, including both newly diagnosed and chronic patients. Integration of EV-miRNA profiles with disease progression, response to treatment and platelet count could help determine their further diagnostic and prognostic value. At the same time, a deeper understanding of the interplay between EV biology and miRNA profiling could facilitate the development of more accurate diagnostic tools and engineered EV-based therapeutic strategies aimed at restoring immune homeostasis in pediatric ITP.

## References

[B1] Alivernini S. Gremese E. McSharry C. Tolusso B. Ferraccioli G. McInnes I. B. (2017). MicroRNA-155-at the critical interface of innate and adaptive immunity in arthritis. Front. Immunology 8, 1932. 10.3389/fimmu.2017.01932 29354135 PMC5760508

[B2] Allegra A. Cicero N. Mirabile G. Giorgianni C. M. Gangemi S. (2023). Novel biomarkers for diagnosis and monitoring of immune thrombocytopenia. Int. J. Mol. Sci. 24 (5), 4438. 10.3390/ijms24054438 PMC10003036 36901864

[B3] Aloi N. Drago G. Ruggieri S. Cibella F. Colombo P. Longo V. (2024). Extracellular vesicles and immunity: at the crossroads of cell communication. Int. J. Mol. Sci. 25 (2), 1205. 10.3390/ijms25021205 PMC10816988 38256278

[B4] Anandagoda N. Willis J. C. Hertweck A. Roberts L. B. Jackson I. Gökmen M. R. (2019). microRNA-142-mediated repression of phosphodiesterase 3B critically regulates peripheral immune tolerance. J. Clin. Invest 129 (3), 1257–1271. 10.1172/JCI124725 30741720 PMC6391082

[B5] Arailym Y. Abaildayev A. Ayaz B. (2026). Extracellular vesicle-derived MicroRNAs as early diagnostic biomarkers of diabetic nephropathy and cardiovascular diseases in type 2 diabetes. Int. J. Mol. Sci. 27 (12), 5581. 10.3390/ijms27125581 PMC13300076 42353293

[B6] Artimovič P. Špaková I. Macejková E. Pribulová T. Rabajdová M. Mareková M. (2024). The ability of microRNAs to regulate the immune response in ischemia/reperfusion inflammatory pathways. Genes Immun. 25 (4), 277–296. 10.1038/s41435-024-00283-6 PMC11327111 38909168

[B7] Avendaño-Portugal C. Montaño-Samaniego M. Guttman-Bazbaz R. Bravo-Estupiñan D. M. Ibáñez-Hernández M. (2025). Therapeutic applications of Poly-miRNAs and miRNA sponges. Int. J. Mol. Sci. 26 (10), 4535. 10.3390/ijms26104535 PMC12111552 40429680

[B8] Barger J. F. Rahman M. A. Jackson D. Acunzo M. Nana-Sinkam S. P. (2016). Extracellular miRNAs as biomarkers in cancer. Food Chem. Toxicol. 98 (Pt A), 66–72. 10.1016/j.fct.2016.06.010 27311798 PMC5086286

[B9] Bay A. Coskun E. Oztuzcu S. Ergun S. Yilmaz F. Aktekin E. (2014). Plasma microRNA profiling of pediatric patients with immune thrombocytopenic purpura. Blood Coagul. Fibrinolysis 25 (4), 379–383. 10.1097/MBC.0000000000000069 24418947

[B10] Bettin B. Gasecka A. Li B. Dhondt B. Hendrix A. Nieuwland R. (2022). Removal of platelets from blood plasma to improve the quality of extracellular vesicle research. J. Thrombosis Haemostasis JTH 20 (11), 2679–2685. 10.1111/jth.15867 PMC9825910 36043239

[B11] Bidgood G. M. Keating N. Doggett K. Nicholson S. E. (2024). SOCS1 is a critical checkpoint in immune homeostasis, inflammation and tumor immunity. Front. Immunology 15, 1419951. 10.3389/fimmu.2024.1419951 PMC11211259 38947335

[B12] Bofill-De Ros X. Vang Ørom U. A. (2024). Recent progress in miRNA biogenesis and decay. RNA Biol. 21 (1), 1–8. 10.1080/15476286.2023.2288741 PMC10761092 38031325

[B13] Bracht J. W. P. Los M. van Eijndhoven M. A. J. Bettin B. van der Pol E. Pegtel D. M. (2023). Platelet removal from human blood plasma improves detection of extracellular vesicle-associated miRNA. J. Extracellular Vesicles 12 (2), e12302. 10.1002/jev2.12302 PMC9929339 36788785

[B14] Bussel J. B. Garcia C. A. (2022). Diagnosis of immune thrombocytopenia, including secondary forms, and selection of second-line treatment. Haematologica 107 (9), 2018–2036. 10.3324/haematol.2021.279513 PMC9425307 35708136

[B15] Buzas E. I. (2023). The roles of extracellular vesicles in the immune system. Nat. Rev. Immunol. 23 (4), 236–250. 10.1038/s41577-022-00763-8 PMC9361922 35927511

[B16] Chang Y. Chen X. Tian Y. Gao X. Liu Z. Dong X. (2020). Downregulation of microRNA-155-5p prevents immune thrombocytopenia by promoting macrophage M2 polarization *via* the SOCS1-dependent PD1/PDL1 pathway. Life Sci. 257, 118057. 10.1016/j.lfs.2020.118057 32634427

[B17] Charlon-Gay J. Nolli S. Dunoyer-Geindre S. Ciepla P. Reny J. L. Fontana P. (2026). MicroRNA-223-3p is a determinant of platelet procoagulant activity. Blood Adv. 10 (1), 83–94. 10.1182/bloodadvances.2024015290 PMC12804390 41056510

[B18] Chen Z. Zhou S. Li J. Li H. Huang C. Guo Q. (2021). SDF-1α facilitates mesenchymal stem cells to induce regulatory B cell differentiation from patients with immune thrombocytopenia. Stem Cells Int. 2021, 3254488. 10.1155/2021/3254488 PMC8592740 34790240

[B19] Chen J. Li J. Yin X. Fu Z. Chen X. Zhang C.-Y. (2026). EV-encapsulated extracellular microRNAs as orchestrators of systemic communication in immunity and inflammation. Immun. and Inflamm. 2 (1), 26. 10.1007/s44466-026-00042-4

[B20] Clark E. A. Kalomoiris S. Nolta J. A. Fierro F. A. (2014). Concise review: MicroRNA function in multipotent mesenchymal stromal cells. Stem Cells 32 (5), 1074–1082. 10.1002/stem.1623 24860868 PMC10668871

[B21] D'Amico G. Starace R. Della Lastra M. Marimpietri D. Dander E. Morandi F. (2025). Dysregulation of the bone marrow microenvironment in pediatric tumors: the role of extracellular vesicles in acute leukemias and neuroblastoma. Int. J. Mol. Sci. 26 (11). 10.3390/ijms26115380 PMC12154240 40508186

[B22] Dang C. P. Leelahavanichkul A. (2020). Over-expression of miR-223 induces M2 macrophage through glycolysis alteration and attenuates LPS-Induced sepsis mouse model, the cell-based therapy in sepsis. PLoS One 15 (7), e0236038. 10.1371/journal.pone.0236038 32658933 PMC7357756

[B23] Despotovic J. M. Grimes A. B. (2018). Pediatric ITP: is it different from adult ITP? Hematol. Am. Soc. Hematol. Educ. Program 2018 (1), 405–411. 10.1182/asheducation-2018.1.405 PMC6246008 30504339

[B24] Di Paola A. Palumbo G. Merli P. Argenziano M. Tortora C. Strocchio L. (2020). Effects of eltrombopag on *in vitro* macrophage polarization in pediatric immune thrombocytopenia. Int. J. Mol. Sci. 22 (1), 97. 10.3390/ijms22010097 PMC7796119 33374151

[B25] Di Paola A. Palumbo G. Tortora C. Argenziano M. Catanoso M. Di Leva C. (2022). Eltrombopag in paediatric immune thrombocytopenia: iron metabolism modulation in mesenchymal stromal cells. Br. Journal Haematology 197 (1), 110–119. 10.1111/bjh.18012 PMC9303225 34961933

[B26] Duan W. Zhang W. Jia J. Lu Q. Eric Gershwin M. (2019). Exosomal microRNA in autoimmunity. Cell Mol. Immunol. 16 (12), 932–934. 10.1038/s41423-019-0319-9 31664221 PMC6884449

[B27] El Zaiat R. S. Abouelenin M. A. H. Saleh A. A. El-Hawy M. Ahmedy I. A. Mansour M. M. (2025). MiR-106b-5p, MiR-200c-3p, and MiR-146a-5p expression as putative biomarkers for disease state in primary immune thrombocytopenia. Expert Rev. Hematol. 18 (8), 689–696. 10.1080/17474086.2025.2522298 40526093

[B28] Gaál Z. (2024). Role of microRNAs in immune regulation with translational and clinical applications. Int. J. Mol. Sci. 25 (3), 1942. 10.3390/ijms25031942 PMC10856342 38339220

[B29] Geekiyanage H. Rayatpisheh S. Wohlschlegel J. A. Brown R. Jr. Ambros V. (2020). Extracellular microRNAs in human circulation are associated with miRISC complexes that are accessible to anti-AGO2 antibody and can bind target mimic oligonucleotides. Proc. Natl. Acad. Sci. U. S. A. 117 (39), 24213–24223. 10.1073/pnas.2008323117 32929008 PMC7533700

[B30] Gilyazova I. Asadullina D. Kagirova E. Sikka R. Mustafin A. Ivanova E. (2023). MiRNA-146a-A key player in immunity and diseases. Int. J. Mol. Sci. 24 (16), 12767. 10.3390/ijms241612767 PMC10454149 37628949

[B31] Gotesman M. Shear M. Raheel S. Procassini M. Panosyan E. H. (2024). Pediatric immune thrombocytopenia. Adv. Pediatr. 71 (1), 229–240. 10.1016/j.yapd.2024.02.007 38944486

[B32] Groot M. Lee H. (2020). Sorting mechanisms for MicroRNAs into extracellular vesicles and their associated diseases. Cells 9 (4). 10.3390/cells9041044 32331346 PMC7226101

[B33] Guo H. Ma J. Zhang Y. Mao Y. Hu Z. Lin Y. (2023). Delivery of AntagomiR-7 through polymer nanoparticles for assisting B cell to alleviate systemic lupus erythematosus. Front. Bioeng. Biotechnol. 11, 1180302. 10.3389/fbioe.2023.1180302 PMC10154577 37152645

[B34] Guo D. Yan J. Yang Z. Chen M. Zhong W. Yuan X. (2024). The immune regulatory role of exosomal miRNAs and their clinical application potential in heart failure. Front. Immunology 15, 1476865. 10.3389/fimmu.2024.1476865 PMC11647038 39687609

[B35] He Y. Ji D. Lu W. Li F. Huang X. Huang R. (2021). Bone marrow mesenchymal stem cell-derived exosomes induce the Th17/Treg imbalance in immune thrombocytopenia through miR-146a-5p/IRAK1 axis. Hum. Cell 34 (5), 1360–1374. 10.1007/s13577-021-00547-7 34052997

[B36] Hua M. Li J. Wang C. Shao L. Hou M. Peng J. (2019). Aberrant expression of microRNA in CD4(+) cells contributes to Th17/Treg imbalance in primary immune thrombocytopenia. Thromb. Res. 177, 70–78. 10.1016/j.thromres.2019.03.005 30856381

[B37] Huang W. Paul D. Calin G. A. Bayraktar R. (2023). miR-142: a master regulator in hematological malignancies and therapeutic opportunities. Cells 13 (1), 84. 10.3390/cells13010084 PMC10778542 38201290

[B38] Huang Z. Zhao X. Wen W. Shi R. Liang G. (2025). Exosome miRNA sorting controlled by RNA-binding protein-motif interactions. Extracell. Vesicles Circ. Nucl. Acids 6 (3), 475–503. 10.20517/evcna.2025.47 PMC12540272 41132505

[B39] Jiao P. Wang X. P. Luoreng Z. M. Yang J. Jia L. Ma Y. (2021). miR-223: an effective regulator of immune cell differentiation and inflammation. Int. J. Biol. Sci. 17 (9), 2308–2322. 10.7150/ijbs.59876 PMC8241730 34239357

[B40] Jolink A. C. Nelson V. S. Schipperus M. R. Amini S. N. Vidarsson G. van der Schoot C. E. (2021). Potential diagnostic approaches for prediction of therapeutic responses in immune thrombocytopenia. J. Clin. Med. 10 (15), 3403. 10.3390/jcm10153403 PMC8347743 34362187

[B41] Kim Y. K. Kim B. Kim V. N. (2016). Re-evaluation of the roles of DROSHA, export in 5, and DICER in microRNA biogenesis. Proc. Natl. Acad. Sci. U. S. A. 113 (13), E1881–E1889. 10.1073/pnas.1602532113 26976605 PMC4822641

[B42] Kumar M. A. Baba S. K. Sadida H. Q. Marzooqi S. A. Jerobin J. Altemani F. H. (2024). Extracellular vesicles as tools and targets in therapy for diseases. Signal Transduct. Target Ther. 9 (1), 27. 10.1038/s41392-024-01735-1 PMC10838959 38311623

[B43] Lee J. M. (2023). Advances in management of pediatric chronic immune thrombocytopenia: a narrative review. J. Yeungnam Med. Sci. 40 (3), 241–246. 10.12701/jyms.2022.00745 PMC10390280 36617702

[B44] Lee I. Choi Y. Shin D. U. Kwon M. Kim S. Jung H. (2023). Small extracellular vesicles as a new class of medicines. Pharmaceutics 15 (2), 325. 10.3390/pharmaceutics15020325 PMC9961868 36839647

[B45] Leng Q. Ding J. Dai M. Liu L. Fang Q. Wang D. W. (2022). Insights into platelet-derived MicroRNAs in cardiovascular and oncologic diseases: potential predictor and therapeutic target. Front. Cardiovasc Med. 9, 879351. 10.3389/fcvm.2022.879351 PMC9218259 35757325

[B46] Li J. Q. Hu S. Y. Wang Z. Y. Lin J. Jian S. Dong Y. C. (2015). MicroRNA-125-5p targeted CXCL13: a potential biomarker associated with immune thrombocytopenia. Am. J. Transl. Res. 7 (4), 772–780. 26064444 PMC4455351

[B47] Li J. Xia Y. Fan X. Wu X. Yang F. Hu S. (2021). Extracellular vesicles derived from miR-199a-5p-modified adipose-derived mesenchymal stem cells alleviate immune thrombocytopenia by inhibiting T helper 17 differentiation. Laboratory Investigation; a Journal Technical Methods Pathology 101 (3), 318–327. 10.1038/s41374-020-00515-z 33402685

[B48] Li Q. Ding Y. Shi Y. Qiu C. Lei L. Li S. (2026). 80 years of extracellular vesicles: from discovery to clinical translation. Extracell. Vesicles Circ. Nucl. Acids 7 (1), 165–233. 10.20517/evcna.2025.161 PMC12902917 41695594

[B49] Lima J. F. Cerqueira L. Figueiredo C. Oliveira C. Azevedo N. F. (2018). Anti-miRNA oligonucleotides: a comprehensive guide for design. RNA Biol. 15 (3), 338–352. 10.1080/15476286.2018.1445959 29570036 PMC5927725

[B50] Liu X. M. Halushka M. K. (2025). Beyond the bubble: a debate on microRNA sorting into extracellular vesicles. Laboratory Investigation; a Journal Technical Methods Pathology 105 (2), 102206. 10.1016/j.labinv.2024.102206 PMC11842217 39647608

[B51] Liu L. Hua M. Liu C. He N. Li Z. Ma D. (2016). The aberrant expression of microRNAs and correlations with T cell subsets in patients with immune thrombocytopenia. Oncotarget 7 (47), 76453–76463. 10.18632/oncotarget.12949 27802180 PMC5363522

[B52] Liu J. Xu Z. Yu J. Zang X. Jiang S. Xu S. (2024). MiR-146a-5p engineered hucMSC-derived extracellular vesicles attenuate dermatophagoides farinae-induced allergic airway epithelial cell inflammation. Front. Immunology 15, 1443166. 10.3389/fimmu.2024.1443166 PMC11446808 39364406

[B53] Liu Z. Rebetz J. Garabet L. Ghanima W. Xu P. Wadenvik H. (2026). MicroRNAs as potential architects of immune dysregulation and megakaryocytic failure in immune thrombocytopenia. Br. Journal Haematology 209 (1), 16–27. 10.1111/bjh.70485 PMC13340496 41983341

[B54] Luisotti L. Germelli L. Piccarducci R. Giacomelli C. Marchetti L. Martini C. (2025). Extracellular vesicles as vehicles for small non-coding RNA therapeutics: standardization challenges for clinical translation. Extracell. Vesicles Circ. Nucl. Acids 6 (3), 403–432. 10.20517/evcna.2025.33 PMC12540276 41132510

[B55] Luque-Campos N. Bustamante-Barrientos F. A. Pradenas C. García C. Araya M. J. Bohaud C. (2021). The macrophage response is driven by mesenchymal stem cell-mediated metabolic reprogramming. Front. Immunology 12, 624746. 10.3389/fimmu.2021.624746 PMC8213396 34149687

[B56] Mahesh G. Biswas R. (2019). MicroRNA-155: a master regulator of inflammation. J. Interferon Cytokine Res. 39 (6), 321–330. 10.1089/jir.2018.0155 30998423 PMC6591773

[B57] Marocco F. Garbo S. Montaldo C. Colantoni A. Quattrocchi L. Gaboardi G. (2025). Negative regulation of miRNA sorting into EVs is mediated by the capacity of RBP PCBP2 to impair the SYNCRIP-Dependent miRNA loading. Elife 14. 10.7554/eLife.105017 PMC12221297 40601477

[B58] Mori M. A. Ludwig R. G. Garcia-Martin R. Brandão B. B. Kahn C. R. (2019). Extracellular miRNAs: from biomarkers to mediators of physiology and disease. Cell Metab. 30 (4), 656–673. 10.1016/j.cmet.2019.07.011 31447320 PMC6774861

[B59] Nelson V. S. Jolink A. C. Amini S. N. Zwaginga J. J. Netelenbos T. Semple J. W. (2021). Platelets in ITP: victims in charge of their own fate? Cells 10 (11), 3235. 10.3390/cells10113235 PMC8621961 34831457

[B60] O'Brien J. Hayder H. Zayed Y. Peng C. (2018). Overview of MicroRNA biogenesis, mechanisms of actions, and circulation. Front. Endocrinol. (Lausanne) 9, 402. 10.3389/fendo.2018.00402 PMC6085463 30123182

[B61] O'Grady T. Njock M. S. Lion M. Bruyr J. Mariavelle E. Galvan B. (2022). Sorting and packaging of RNA into extracellular vesicles shape intracellular transcript levels. BMC Biol. 20 (1), 72. 10.1186/s12915-022-01277-4 PMC8944098 35331218

[B62] Olson W. J. Derudder E. (2023). The miR-142 miRNAs: shaping the naïve immune system. Immunol. Lett. 261, 37–46. 10.1016/j.imlet.2023.07.005 37459958

[B63] Palviainen M. Puutio J. Østergaard R. H. Eble J. A. Maaninka K. Butt U. (2024). Beyond basic characterization and omics: immunomodulatory roles of platelet-derived extracellular vesicles unveiled by functional testing. J. Extracellular Vesicles 13 (10), e12513. 10.1002/jev2.12513 PMC11428872 39330919

[B64] Payandeh Z. Tangruksa B. Synnergren J. Heydarkhan-Hagvall S. Nordin J. Z. Andaloussi S. E. (2024). Extracellular vesicles transport RNA between cells: unraveling their dual role in diagnostics and therapeutics. Mol. Asp. Med. 99, 101302. 10.1016/j.mam.2024.101302 39094449

[B65] Pereira M. Gazzinelli R. T. (2023). Regulation of innate immune signaling by IRAK proteins. Front. Immunology 14, 1133354. 10.3389/fimmu.2023.1133354 PMC9972678 36865541

[B66] Provan D. Arnold D. M. Bussel J. B. Chong B. H. Cooper N. Gernsheimer T. (2019). Updated international consensus report on the investigation and management of primary immune thrombocytopenia. Blood Adv. 3 (22), 3780–3817. 10.1182/bloodadvances.2019000812 31770441 PMC6880896

[B67] Puhm F. Boilard E. Machlus K. R. (2021). Platelet extracellular vesicles: beyond the blood. Arterioscler. Thromb. Vasc. Biol. 41 (1), 87–96. 10.1161/ATVBAHA.120.314644 PMC7769913 33028092

[B68] Qian B.-H. Ye X. Zhang L. Sun Y. Zhang J.-R. Gu M.-L. (2014). Increased miR-155 expression in peripheral blood mononuclear cells of primary immune thrombocytopenia patients was correlated with serum cytokine profiles. Acta Haematol. 133 (3), 257–263. 10.1159/000362150 25413124

[B69] Qian C. Yan W. Li T. Cui Q. Liu P. Gu M. (2018). Differential expression of MiR-106b-5p and MiR-200c-3p in newly diagnosed *versus* chronic primary immune thrombocytopenia patients based on systematic analysis. Cell Physiol. Biochem. 45 (1), 301–318. 10.1159/000486811 29402802

[B70] Qu M. Zou X. Fang F. Wang S. Xu L. Zeng Q. (2020). Platelet-derived microparticles enhance megakaryocyte differentiation and platelet generation *via* miR-1915-3p. Nat. Commun. 11 (1), 4964. 10.1038/s41467-020-18802-0 33009394 PMC7532443

[B71] Rajendran R. L. Gangadaran P. (2026). Mesenchymal stem cells and extracellular vesicles: bridging the translational gap in regenerative medicine. Int. J. Mol. Sci. 27 (12), 5423. 10.3390/ijms27125423 PMC13299713 42353140

[B72] Rong Y. Lu W. Huang X. Ji D. Tang D. Huang R. (2024). Exosomal miR-146a-5p derived from bone marrow mesenchymal stromal cells regulate Th1/Th2 balance and alleviates immune thrombocytopenia in pregnancy. Hum. Cell 38 (1), 31. 10.1007/s13577-024-01162-y 39699695

[B73] Rusca N. Monticelli S. (2011). MiR-146a in immunity and disease. Mol. Biol. Int. 2011, 437301. 10.4061/2011/437301 22091404 PMC3200075

[B74] Russo G. Parodi E. Farruggia P. Notarangelo L. D. Perrotta S. Casale M. (2024). Recommendations for the management of acute immune thrombocytopenia in children. A consensus conference from the Italian association of pediatric hematology and oncology. Blood Transfus. 22 (3), 253–265. 10.2450/BloodTransfus.501 PMC11073630 37677093

[B75] Seto A. G. Beatty X. Lynch J. M. Hermreck M. Tetzlaff M. Duvic M. (2018). Cobomarsen, an oligonucleotide inhibitor of miR-155, co-ordinately regulates multiple survival pathways to reduce cellular proliferation and survival in cutaneous T-cell lymphoma. Br. Journal Haematology 183 (3), 428–444. 10.1111/bjh.15547 30125933

[B76] Seyhan A. A. (2024). Trials and tribulations of MicroRNA therapeutics. Int. J. Mol. Sci. 25 (3), 1469. 10.3390/ijms25031469 PMC10855871 38338746

[B77] Shah H. Liu Z. Guo W. Ren W. Xiao Y. (2025). Immune-regulating extracellular vesicles: a new frontier in autoimmune disease therapy. Essays Biochemistry 69 (2), 161–168. 10.1042/ebc20253016 PMC12224888 40366303

[B78] Shahryari F. Jafarinia M. Jafarinia M. Azimzadeh M. (2025). Immunomodulatory and neuroprotective effects of miR-146a-enriched MSC-Derived extracellular vesicles in experimental autoimmune encephalomyelitis. Int. Immunopharmacol. 166, 115584. 10.1016/j.intimp.2025.115584 40991997

[B79] Sharma S. (2017). Immunomodulation: a definitive role of microRNA-142. Dev. Comp. Immunol. 77, 150–156. 10.1016/j.dci.2017.08.001 28801229

[B80] Shi B. Phan T. K. Poon I. K. H. (2025). Extracellular vesicles from the dead: the final message. Trends Cell Biol. 35 (5), 439–452. 10.1016/j.tcb.2024.09.005 39438206

[B81] Singh A. Uzun G. Bakchoul T. (2021). Primary immune thrombocytopenia: novel insights into pathophysiology and disease management. J. Clin. Med. 10 (4), 789. 10.3390/jcm10040789 PMC7920457 33669423

[B82] Stone A. P. Nikols E. Freire D. Machlus K. R. (2022). The pathobiology of platelet and megakaryocyte extracellular vesicles: a (C)lot has changed. J. Thrombosis Haemostasis JTH 20 (7), 1550–1558. 10.1111/jth.15750 35506218

[B83] Surman M. Stępień E. Hoja-Łukowicz D. Przybyło M. (2017). Deciphering the role of ectosomes in cancer development and progression: focus on the proteome. Clin. Exp. Metastasis 34 (3-4), 273–289. 10.1007/s10585-017-9844-z 28317069 PMC5442264

[B84] Tan J. H. Ahmad Azahari A. H. S. Ali A. Ismail N. A. S. (2023). Scoping review on epigenetic mechanisms in primary immune thrombocytopenia. Genes (Basel) 14 (3), 555. 10.3390/genes14030555 PMC10048672 36980827

[B85] Thakur Y. Meshram R. J. Taksande A. (2024). Diagnosis and management of immune thrombocytopenia in paediatrics: a comprehensive review. Cureus 16 (9), e69635. 10.7759/cureus.69635 PMC11488990 39429356

[B86] Villar-Vesga J. Grajales C. Burbano C. Vanegas-García A. Muñoz-Vahos C. H. Vásquez G. (2019). Platelet-derived microparticles generated *in vitro* resemble circulating vesicles of patients with rheumatoid arthritis and activate monocytes. Cell Immunol. 336, 1–11. 10.1016/j.cellimm.2018.12.002 30538031

[B87] Villarroya-Beltri C. Gutiérrez-Vázquez C. Sánchez-Cabo F. Pérez-Hernández D. Vázquez J. Martin-Cofreces N. (2013). Sumoylated hnRNPA2B1 controls the sorting of miRNAs into exosomes through binding to specific motifs. Nat. Commun. 4, 2980. 10.1038/ncomms3980 24356509 PMC3905700

[B88] Wang D. Tang M. Zong P. Liu H. Zhang T. Liu Y. (2018). MiRNA-155 regulates the Th17/Treg ratio by targeting SOCS1 in severe acute pancreatitis. Front. Physiology. 9–2018. 10.3389/fphys.2018.00686 PMC6002743 29937734

[B89] Wang Y. Zhang J. Su Y. Wang C. Zhang G. Liu X. (2020). miRNA-98-5p targeting IGF2BP1 induces mesenchymal stem cell apoptosis by modulating PI3K/Akt and p53 in immune thrombocytopenia. Mol. Ther. Nucleic Acids 20, 764–776. 10.1016/j.omtn.2020.04.013 32428701 PMC7232042

[B90] Wang Y. Guo Y. Zhang X. Zhao H. Zhang B. Wu Y. (2021). The role and mechanism of miR-557 in inhibiting the differentiation and maturation of megakaryocytes in immune thrombocytopenia. RNA Biol. 18 (11), 1953–1968. 10.1080/15476286.2021.1884783 PMC8582991 33586614

[B91] Wang W. L. Ouyang C. Graham N. M. Zhang Y. Cassady K. Reyes E. Y. (2022). microRNA-142 guards against autoimmunity by controlling treg cell homeostasis and function. PLoS Biol. 20 (2), e3001552. 10.1371/journal.pbio.3001552 PMC8893712 35180231

[B92] Wang Y. Liu C. Hu Y. Sun X. Zhang W. Zhu W. (2026a). MPL receptor dimerization and the thrombopoietin pathway in primary immune thrombocytopenia: from molecular mechanisms to targeted therapies. Front. Medicine 13, 1798504. 10.3389/fmed.2026.1798504 PMC13283879 42338948

[B93] Wang Z. Dai X. Li P. Ma J. Ma Y. Tang Y. (2026b). Association and exploratory clinical significance of peripheral blood mononuclear cell microRNA-155 with T-Cell subset imbalance in newly diagnosed paediatric immune thrombocytopenia. BMC Pediatrics 26 (1), 816. 10.1186/s12887-026-07360-x PMC13532002 42681618

[B94] Welsh J. A. Goberdhan D. C. I. O'Driscoll L. Buzas E. I. Blenkiron C. Bussolati B. (2024). Minimal information for studies of extracellular vesicles (MISEV2023): from basic to advanced approaches. J. Extracellular Vesicles 13 (2), e12404. 10.1002/jev2.12404 PMC10850029 38326288

[B95] Wu Y. Li X. Jin Y. Mao F. Zhang L. (2026). Exosomal non-coding RNAs in autoimmune diseases: molecular mechanisms and potential applications. Front. Immunology 17, 1753611. 10.3389/fimmu.2026.1753611 PMC12960517 41798919

[B96] Xie G.-Y. Song D. Luo T. Liao Y. Guo A.-Y. Lei Q. (2026). EVmiRNA2.0: an updated database for miRNA expression in comprehensive human extracellular vesicles. Nucleic Acids Res. 54 (D1), D128–D134. 10.1093/nar/gkaf1054 41157914 PMC12807601

[B97] Xu D. Di K. Fan B. Wu J. Gu X. Sun Y. (2022). MicroRNAs in extracellular vesicles: sorting mechanisms, diagnostic value, isolation, and detection technology. Front. Bioeng. Biotechnol. 10, 948959. 10.3389/fbioe.2022.948959 36324901 PMC9618890

[B98] Xu Q. Hou W. Zhao B. Fan P. Wang S. Wang L. (2024). Mesenchymal stem cells lineage and their role in disease development. Mol. Med. 30 (1), 207. 10.1186/s10020-024-00967-9 PMC11552129 39523306

[B99] Yáñez-Mó M. Siljander P. R. Andreu Z. Zavec A. B. Borràs F. E. Buzas E. I. (2015). Biological properties of extracellular vesicles and their physiological functions. J. Extracellular Vesicles 4, 27066. 10.3402/jev.v4.27066 25979354 PMC4433489

[B100] Yang X. Hong S. Yan T. Liu M. Liu M. Zhao J. (2025). MiR-146a engineered extracellular vesicles derived from mesenchymal stromal cells more potently attenuate ischaemia-reperfusion injury in lung transplantation. Clin. Transl. Med. 15 (4), e70298. 10.1002/ctm2.70298 40195092 PMC11975614

[B101] Yao R. Ma Y. L. Liang W. Li H. H. Ma Z. J. Yu X. (2012). MicroRNA-155 modulates treg and Th17 cells differentiation and Th17 cell function by targeting SOCS1. PLoS One 7 (10), e46082. 10.1371/journal.pone.0046082 23091595 PMC3473054

[B102] Zhang J. Wang L. Shen Y. Qian X. Wang Z. Wang C. (2025). miR-223 alleviates DSS-Induced colitis by prompting macrophage M2 polarization through PPAR-γ/FOXO1 signaling. Front. Immunology 16, 1598781. 10.3389/fimmu.2025.1598781 PMC12339350 40799643

[B103] Zhao Y. Cui S. Wang Y. Xu R. (2022). The extensive regulation of MicroRNA in immune thrombocytopenia. Clin. Appl. Thromb. Hemost. 28, 10760296221093595. 10.1177/10760296221093595 35536600 PMC9096216

[B104] Zhou X. Shan N. (2026). Innovative strategies for immune thrombocytopenia treatment: immunomodulatory mechanisms and clinical potential of mesenchymal stem cells. Stem Cell Res. and Ther. 17 (1), 189. 10.1186/s13287-026-05000-w PMC13188697 41947170

[B105] Zhu H. Ruan X. Zhao K. Kuang W. Liu S. Yan W. (2024). The miR-641-STIM1 and SATB1 axes play important roles in the regulation of the Th17/Treg balance in ITP. Sci. Rep. 14 (1), 11243. 10.1038/s41598-024-61660-9 38755179 PMC11098809

[B106] Żmigrodzka M. Guzera M. Miśkiewicz A. Jagielski D. Winnicka A. (2016). The biology of extracellular vesicles with focus on platelet microparticles and their role in cancer development and progression. Tumour Biol. 37 (11), 14391–14401. 10.1007/s13277-016-5358-6 27629289 PMC5126185

